# Comment on ‘Total Magnesium Intake and Risk of Frailty in Older Women’ by Struijk Et Al.

**DOI:** 10.1002/jcsm.13592

**Published:** 2024-10-03

**Authors:** Long Guo, Qing Lan, Ming Zhou, Fei Liu

**Affiliations:** ^1^ Department of Urology The Second Affiliated Hospital of Nanchang University Nanchang China; ^2^ Department of Gastroenterology, Liyuan Hospital Affiliated to Tongji Medical College Huazhong University of Science and Technology Wuhan China

To Editor,

We recently read an article by Struijk et al. [1] published in the *Journal of Cachexia, Sarcopenia and Muscle* entitled ‘Total Magnesium Intake and Risk of Frailty in Older Women’. This study provides valuable insight into the relationship between magnesium intake and risk of frailty in older women, with particular emphasis on the importance of dietary magnesium. I commend the authors for their thorough analysis and use of a large cohort study spanning several decades, which greatly strengthens the findings. However, there are a few points that I think deserve further discussion in order to round out this important study.

Firstly, in the article, the authors considered the effect of supplement use on the results of the study and adjusted for the use of magnesium supplements in the statistical analyses. However, there are several factors that need further consideration here that may affect the robustness of the study results. In the study, the data spanned a large period of time (1984 to 2010) and significant changes in healthcare and socio‐economic conditions during this period may have introduced heterogeneity in the results. In addition, the formulations, brands and usage of supplements on the market may have changed over time and there may be instances where actual intake cannot be accurately captured. Future studies could therefore use mixed effects modelling or time series analysis to mitigate the impact of time variation on the results.

In addition, although the authors have adjusted for a variety of confounders, such as lifestyle, medication use and dietary factors, there are still a number of potential confounders that could have an impact on the robustness of the results. For example, previous studies have suggested that supplement users may already have certain health problems or concerns about future health [2], which may motivate them to use supplements more frequently. It is therefore difficult to determine whether the frailty outcomes are due to the effects of the supplements or to these underlying health problems. In addition, mental health status [3], social support [4] or environmental factors [5] may also influence the onset and progression of frailty. Future studies could consider using stratified analyses or taking these potential factors into account as covariates. Exploring these factors in depth would help to better understand the observed differences and provide stronger support for the reliability of the findings.

This also emphasizes the important role of social workers in the research and prevention of frailty syndromes, particularly in the older population. Through direct assistance, community involvement and support, and health promotion as well as lectures, social workers provide comprehensive support to older people to help them stay healthy and delay or prevent the onset of frailty. In addition, social workers work closely with professionals in the medical, nutritional and psychological fields to ensure that older persons receive comprehensive health management. This interdisciplinary collaboration allows for more effective prevention and management of frailty syndromes and improves the overall effectiveness of interventions.

In conclusion, the study by Struijk et al. provides an important foundation for a deeper understanding of the association between magnesium intake and frailty risk in older women [1]. However, to further enhance the accuracy and broad applicability of this study, we suggest that future research should place a greater emphasis on the temporal effects of supplement use and other potential confounders. At the same time, we also emphasize the key role of social workers in tackling frailty syndromes, who, through multilevel support and interdisciplinary collaboration, can significantly improve the health status and quality of life of older people. We believe that these recommendations will help future studies to achieve more comprehensive results in revealing the relationship between magnesium intake and frailty.

## Conflicts of Interest

The authors declare no conflicts of interest.
